# Mid-infrared wavelength multiplexers on an InP platform

**DOI:** 10.1515/nanoph-2024-0756

**Published:** 2025-04-24

**Authors:** Kevin Zhang, Rudolf Mayer, Dominik Burghart, Gerhard Boehm, Mikhail A. Belkin

**Affiliations:** Walter Schottky Institute, Technical University of Munich, Garching 85748, Germany

**Keywords:** integrated photonics, directional coupler, photonic integrated circuit

## Abstract

We demonstrate mid-infrared multiplexers based on evanescent couplers in In_0.53_Ga_0.47_As/InP ridge waveguides. Multiplexing of *λ* = 5.2 µm and *λ* = 8 µm input wavelengths in TM_00_ modes to a single TM_00_ output was achieved with 0.7 dB insertion loss. The demonstrated multiplexing bandwidth is significantly broader than is achievable using typical arrayed waveguide gratings, while displaying comparable insertion loss. These devices will be essential toward the development of broadband multi-color mid-infrared photonic integrated circuits for multi-species gas sensing and multi-band mid-infrared free-space communications.

## 1 Introduction

Integrating photonic components onto a singular chip is crucial for condensing bulky free-space optical setups into portable mass-producible solutions. Photonic integrated circuit (PIC) technologies in the mid-infrared (mid-IR, *λ* ≈ 3–25 µm) spectral range are highly sought after for spectroscopic and sensing applications [1], [2], [3], [4], [5], owing to the numerous molecular vibrational “fingerprint” absorption lines found in this region. Additionally, mid-IR light is advantageous for free space communication [6], [7], [8], [9] as it offers longer propagation distances compared to shorter wavelengths, particularly in adverse weather conditions [10], [11].

The predominant semiconductor laser source in the mid-IR is the quantum cascade laser (QCL), providing room temperature high power output within the entire mid-IR spectral range [12], [13], [14], [15]. We have recently experimentally demonstrated the operation of an InP-based mid-IR photonic integrated circuit (PIC) platform based on the monolithic integration of InGaAs/AlInAs/InP QCLs with In_0.53_Ga_0.47_As/InP passive waveguides [16], [17]. We have also demonstrated that the passive In_0.53_Ga_0.47_As/InP ridge waveguides can be fabricated to possess optical losses as low as 0.5 dB/cm at 5 µm wavelength, and lower than 3 dB/cm up to 10 µm wavelength [4]. Other groups reported similar or even lower mid-IR losses on this platform [18], [19]. This makes the InP platform an ideal materials system to develop broadband mid-IR PICs.

In many instances, one desires to multiplex the outputs of distributed feedback (DFB) QCLs operating at widely spaced wavelengths into a single beam. Examples of applications for such devices include multi-species gas sensing [20] or multi-band free-space communications [21]. It has been shown that the active region of QCLs can combine multiple laser gain sections in a single stack to produce broadly-tunable [22] and multi-color [23], [24], [25], [26] lasers. The emitted light of multiple QCLs on one chip with different emission wavelengths could then be combined to a singular output waveguide using a passive wavelength multiplexer integrated monolithically on the same InP chip.

Wavelength division multiplexing is typically done using arrayed waveguide gratings (AWGs). This architecture allows for multiplexing of a dense arrangement of wavelengths within a narrowband spectral range which is ideal for telecommunications applications. Multiplexing using AWGs has also been demonstrated in the mid-IR recently at *λ* ≈ 5.2 µm [27]. However, for spectroscopic applications, sometimes a more sparse arrangement of wavelengths may be required, but within a very broad spectral band. Additionally, applications such as remote sensing or free space communications often aim to utilize the atmospheric transparency windows at both 3–5 μm and 8–12 μm bands. To this end, here we use In_0.53_Ga_0.47_As/InP waveguiding platform to experimentally demonstrate wavelength division multiplexers using evanescent waveguide couplers [28], [29], capable of multiplexing *λ* ≈ 5.2 and *λ* ≈ 8 µm with an insertion loss of around 0.7 dB. While only two wavelengths were multiplexed in this work, additional evanescent couplers may be independently designed for other wavelengths and fabricated in series to combine an arbitrary number of inputs. Our results indicate that this multiplexing scheme on an InP platform holds much promise for the future development of PICs in the mid-IR.

## 2 Multiplexer design

**Figure 1:**
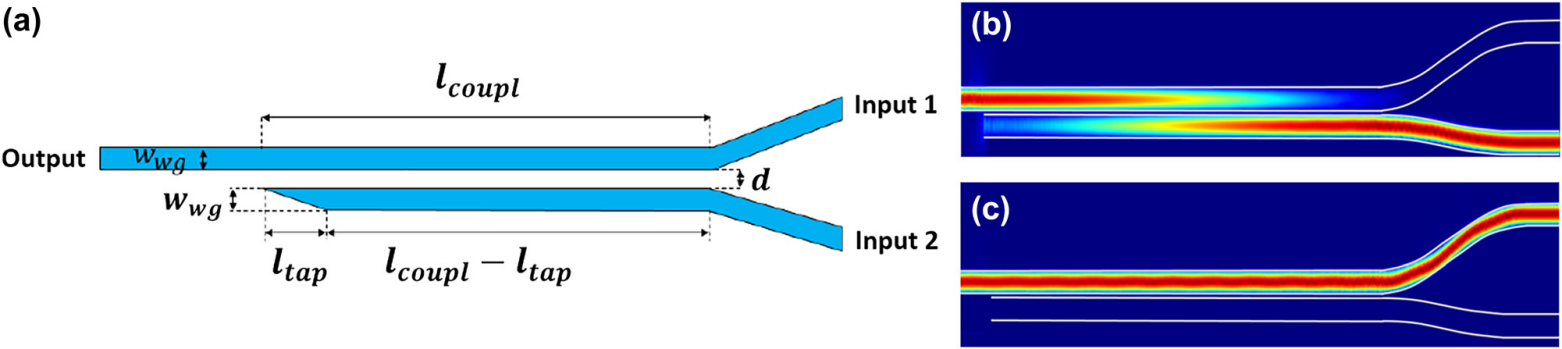
A 2 x 1 evanescent coupler. (a) Schematic. (b) Simulated propagation of a *λ* = 8 μm wave through an evanescent coupling region designed for 100 % power transfer to the upper waveguide. (c) Simulated propagation of a *λ* = 5.2 μm wave passing through the same coupling region unimpeded.

Simulations of waveguide geometries were performed using a finite difference time domain (FDTD) solver, Ansys Lumerical. Given the transverse magnetic (TM) output of QCLs, simulations and experimental measurements reported here focused on TM-polarized modes. The refractive index data for all materials was characterized based on reflectance measurements of thin films grown in house using molecular beam epitaxy (MBE). Propagation losses of these materials has been assumed to be identical to those shown in Ref. [4] due to both samples being grown in the same MBE in house and fabricated with identical fabrication procedures. A pair of identical waveguides are then simulated together with a variable separation, *d*, as shown in Figure 1. At a small enough separation, these two waveguides become coupled, causing a periodic transfer of power between the two. An eigensolver is used to calculate the coupled symmetric and anti-symmetric modes. The index difference between this pair of fundamental coupled modes for every wavelength can be used to determine the period of power transfer between the waveguides using the following expression [30]:

(1) $$P_{\text{out}}\left(L\right)=P_{in}\text{si}{\text{n}}^{2}\left(\frac{\pi L\Delta n\left(\lambda \right)}{\lambda }\right),$$

where *P*_out_ is the power in the secondary waveguide, *P*_in_ is the power in the origin waveguide, *L* is the coupling length, Δ*n* is the effective refractive index difference between the symmetric and anti-symmetric coupled modes, and *λ* is the wavelength. To minimize insertion loss due to optical absorption, the coupler length is minimized while maintaining 100 % power transfer into the secondary waveguide.

**Figure 2:**
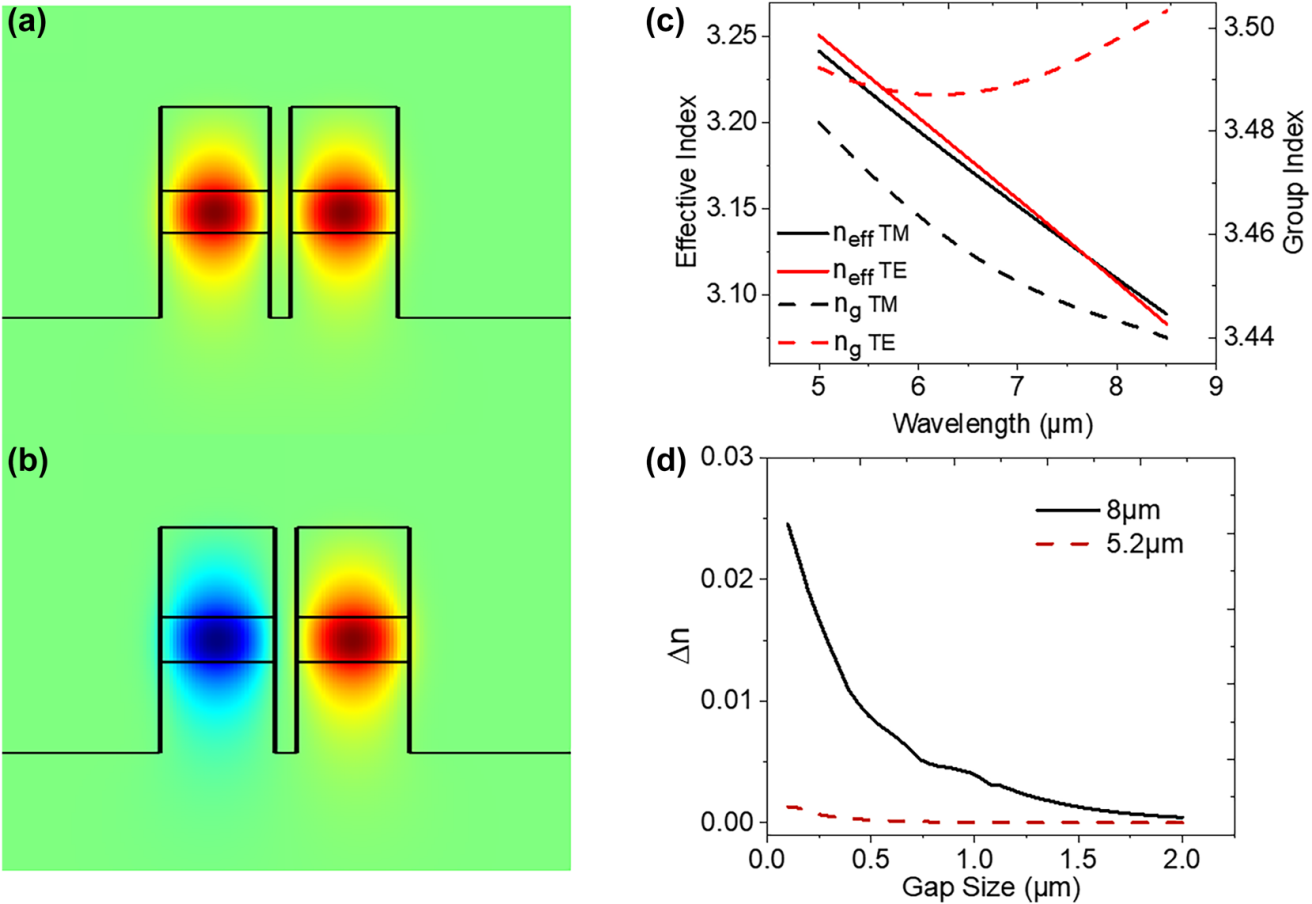
Simulated (a) symmetric and (b) antisymmetric mode for a parallel propagating waveguide pair with 1.5 μm In_0.53_Ga_0.47_As core thickness, 3 μm InP:Fe upper and lower cladding, 5 μm width, and 1 μm separation at *λ* = 8 μm. (c) Simulated values of the effective refractive index and group index for the given waveguide geometry for both the fundamental TE and TM modes. (d) Simulated values of Δ*n* for the same coupled waveguide structure as a function of separation distance for both *λ* = 5.2 μm and 8 μm.

The coupling strength between the two waveguides, as represented by Δ*n*, is shown in Figure 2d for this particular waveguide geometry for different separation distances for both *λ* = 5.2 μm and 8 μm wavelengths. In this method of wavelength multiplexing, the large spectral separation in the wavelengths to be multiplexed is taken advantage of in order to reduce cross talk between channels. Due to the larger mode size of the longer wavelength mode, a waveguide geometry can be chosen such that the shorter wavelength experiences virtually no coupling, while the long wavelength experiences strong coupling. This can be seen in Figure 2d, where the Δ*n* of the *λ* = 5.2 μm mode stays near zero, while the Δ*n* of the *λ* = 8 μm is at least two orders of magnitude larger for waveguide separation distances below 1 μm. As a result, the channel with the shorter wavelength continues unimpeded to the waveguide output facet, while the longer wavelength is multiplexed into the other channel, as shown in Figure 1b and c. When cascading more than one evanescent coupler together, wavelengths must be multiplexed in order of increasing wavelength to avoid channel crosstalk. To achieve sufficient mode coupling while maintaining adequate mode confinement, the waveguide was constructed with a 1.5-μm-thick undoped In_0.53_Ga_0.47_As core, surrounded on top and bottom by 3-μm-thick cladding layers of undoped InP. A waveguide width of 5 μm was chosen. Figure 2c presents the computed effective refractive index and group index for the fundamental TE and TM mode as a function of the mid-IR wavelength for ridge-waveguides with this geometry. The calculated symmetric and antisymmetric mode profiles for this geometry is shown in Figure 2a and b for *λ* = 8 μm. Due to fabrication constraints, a waveguide separation narrower than 1 μm can pose significant challenges. Therefore, the minimum gap size of *d* = 1 μm was chosen, and a coupling length of *L* = 430 μm was calculated.

## 3 Experimental section

### 3.1 Device fabrication

The material layers for the waveguide structure are epitaxially grown on a semi-insulating InP:Fe substrate. The iron compensation significantly reduces the optical losses of the material by trapping free carriers [31]. This substrate is used as the lower cladding of the ridge waveguide structure. On top of the substrate, a layer of nominally undoped lattice-matched In_0.53_Ga_0.47_As with a background doping concentration of *n* = 7 × 10^14^ cm^−3^ is grown with molecular beam epitaxy (MBE). Finally, another layer of nominally undoped InP with a background doping of *n* = 5 × 10^15^ cm^−3^ is grown with MBE on top as the upper cladding of the three-layered waveguide. These materials are all capable of being homogeneously integrated with QCLs and are transparent for the desired mid-IR region. Background carrier concentrations in the core and cladding layers were computed to produce smaller than 0.5 dB/cm propagation loss [4].

Devices are defined using electron beam lithography. The mask pattern was transferred to a Si_*x*_N_*y*_ hard mask using a CF_4_/O_2_ plasma in an inductively coupled plasma reactive ion etching system (ICP-RIE). This was then transferred into the semiconductor layers using a CH_4_/H_2_ plasma in an ICP-RIE. Residual hard mask was removed using another CF_4_/O_2_ plasma etch. Waveguide facets were cleaved to expose input and output facets for external in and out-coupling of light. A multi-layer antireflection coating composed of alternating YF_3_ and ZnS layers was then evaporated onto both facets in order to suppress Fabry–Perot oscillations of the waveguides when measuring with narrow linewidth laser sources.

**Figure 3:**
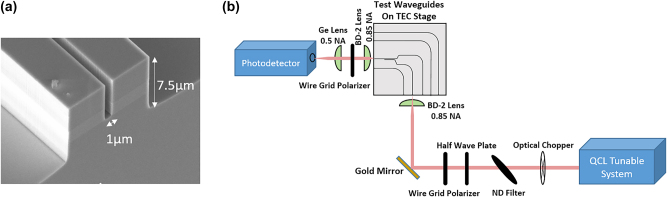
Characterization of fabricated multiplexers. (a) Cross sectional SEM image of the coupling region of an evanescent coupler. The separation gap between the two parallel running waveguides is etched only to the bottom of the In_0.53_Ga_0.47_As core (lighter grey color in the image), while the outside of the waveguides is etched 3 μm deep into InP:Fe substrate. (b) Schematic of the experimental setup used to characterize the evanescent couplers.

Figure 3a shows a cross sectional scanning electron microscope (SEM) image of a device which has been cleaved through the coupling region. It can be seen that, due to a difference in the mass flow in RIE, the outsides of the waveguides have different etch depths compared to the coupling region. This fabrication imperfection results in a small amount of the transmitted optical power at 8 μm wavelength being in the TE-rather than TM-polarized mode as described in the following.

### 3.2 Device characterization

The experimental setup used to characterize the evanescent couplers is shown in Figure 3b. Based on measurements of the Fabry–Perot resonances of similar waveguides as a function of sample temperature, we estimate the effective thermo-optic coefficient of our waveguides to be approximately $\frac{dn_{\text{eff}}}{dT}\approx 3.6\;*\;10^{-4}\;{\text{K}}^{-1}$ . In order to prevent thermo-optic tuning, samples were mounted onto a temperature-controlled stage and stabilized to 20 °C. Light from a broadly tunable QCL laser system (tuning range *λ* = 5.2 μm–6 μm, 7.4 μm–8.5 μm) was coupled to the fundamental TM mode of the waveguides through the input facet using a lens with a numerical aperture (NA) of 0.85 following the approach described in Ref. [4]. Input alignment is carried out using a three axis piezoelectric stage with a nominal accuracy of 20 nm. Light from the output facet was collected and collimated using a lens pair and then focused onto a calibrated nitrogen-cooled mercury-cadmium-telluride (MCT) photodetector. A polarizer within the lens pair ensures measurement of only a single polarization of light from the sample output facet.

Each device is composed of two input and one output waveguide. Both input waveguides have an input facet to allow for external light coupling. One of the input waveguides terminates with a taper at the end of the evanescent coupler and the other continues to the output facet of the sample, as shown in Figure 1b. A 90-degree bend is placed before the evanescent coupler, to prevent light not coupled to the waveguide from reaching the detector, as shown in the schematic of the sample in Figure 3b. The sample also contains continuous 90-degree-bent reference waveguides with the same dimensions and the same input tapers as the waveguides used in the coupler as shown in Figure 3b.

Light was first coupled into a reference waveguide adjacent to the coupler device inputs. The transmission through this waveguide was used as a reference in order to approximately normalize out the losses due to material absorption and scattering. While this method of normalization may be subject to inconsistencies with alignment and coupling efficiency, through repetitive alignment to a singular waveguide, we estimate we are able to achieve identical in/out-coupling efficiency to a waveguide with a standard deviation of the total insertion loss of approximately 5 %.

## 4 Results and discussion

**Figure 4:**
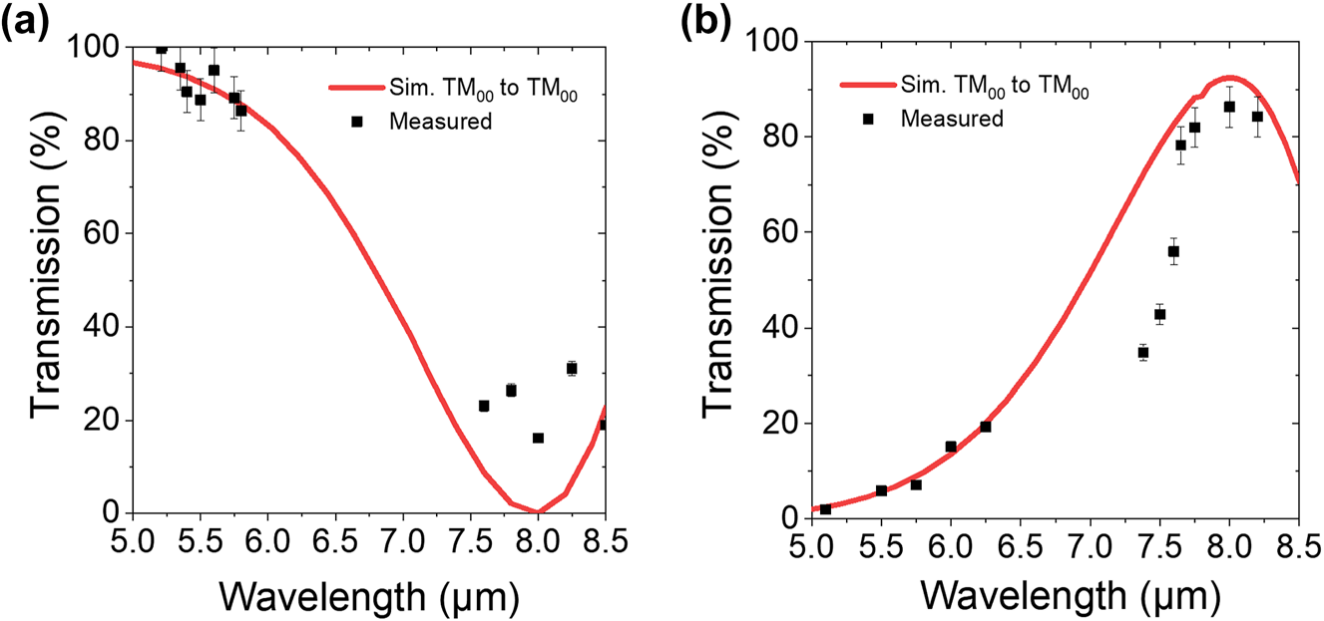
Simulated and measured transmission characteristics of the fabricated evanescent coupler. Total coupler transmission as a function of input wavelength on the (a) *λ* = 5.2 μm input branch and the (b) *λ* = 8 μm branch.

The results of the measured coupler performance versus simulated results are shown in Figure 4, which shows the transmitted optical power through the coupler as a function of wavelength. Several wavelengths within the tuning range of the input laser were tested for both inputs. At the design wavelength of 5.2 μm, the multiplexers achieve a transmitted efficiency of nearly 100 %. The multiplexer is relatively broadband, with a 3 dB cutoff wavelength at *λ*_3dB_ ≈ 7 μm. Similarly, in the 8 μm channel, the multiplexers exhibit a transmitted efficiency of approximately 86 %, or an insertion loss of 0.7 dB with a 3 dB cutoff wavelength also at approximately 7 μm.

**Figure 5:**
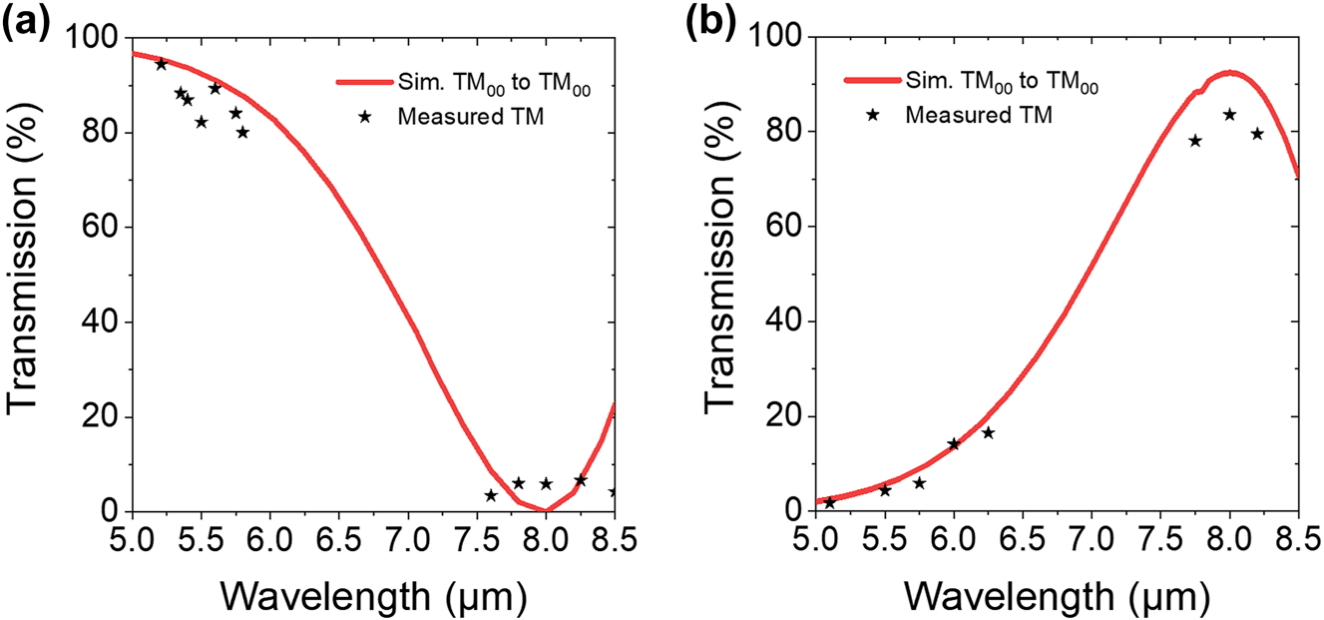
Simulated and measured TM_00_ polarized coupler transmission as a function of input wavelength on the (a) *λ* = 5.2 μm input branch and the (b) *λ* = 8 μm branch.

As mentioned earlier, due to incomplete etching of the gap between the two waveguides in the coupling section of our device, we expect a small amount of polarization conversion for the optical power sent into the 8 μm input of the coupler. Simulations show that this small amount of polarization-converted signal will be coupled to the TE_00_ mode in the output waveguide, while coupling to higher-order TE and TM modes are not expected to play a significant role. By inserting a polarizer at the output of the waveguide, as shown in Figure 3, we can isolate the output from any cross polarization. This new data is shown in Figure 5a and b in comparison with simulation results. Additionally, by utilizing a taper at the end of the evanescent coupler we can ensure minimal back reflection, avoiding any disruptive optical feedback toward the light source.

Compared to the arrayed waveguide gratings demonstrated at *λ* ≈ 5.2 µm in the same material system [27], coupling losses were comparable at ∼0.9 dB. However, as stated previously, the arrayed waveguide grating method of multiplexing is more suitable for combining wavelengths within a relatively narrow spectral band. Conversely, evanescent coupling allows us to combine discrete wavelengths that can be spaced arbitrarily far apart, allowing for photonic integrated chips capable of targeting spectrally distinct gas absorption lines or atmospheric transparency windows spread throughout the entire mid-infrared spectral range on a single chip.

## 5 Conclusions

In summary, we report the first mid-IR waveguide multiplexer based on evanescent couplers on an InP/InGaAs based platform which are capable of combining wavelengths as broadly spaced as *λ* = 8 μm and 5.2 μm, with an efficiency of over 86 %. The efficiency of these devices paired together with the compatibility of this material system with the predominant QCL architecture, opens the door for the production of multi-color mid-IR photonic integrated circuits for applications such as multi-species gas sensing or multi-band free-space communications or light detection and ranging.
